# *Danio Rerio* as Model Organism for Adenoviral Vector Evaluation

**DOI:** 10.3390/genes10121053

**Published:** 2019-12-17

**Authors:** Paola Gulías, Jorge Guerra-Varela, Manuela Gonzalez-Aparicio, Ana Ricobaraza, Africa Vales, Gloria Gonzalez-Aseguinolaza, Rubén Hernandez-Alcoceba, Laura Sánchez

**Affiliations:** 1 Department of Zoology, Genetics and Physical Anthropology, Universidade de Santiago de Compostela, 27002 Lugo, Spain; paola.gulias@rai.usc.es (P.G.); jorge.guerra@usc.es (J.G.-V.); 2 Gene Therapy and Regulation of Gene Expression Program, CIMA, FIMA, University of Navarra, Instituto de Investigación Sanitaria de Navarra, IDISNA, Pamplona, 31009 Navarra, Spain; gamanuela@unav.es (M.G.-A.); aricobaraza@unav.es (A.R.); avales@unav.es (A.V.); ggasegui@unav.es (G.G.-A.); rubenh@unav.es (R.H.-A.); 3 Vivet Therapeutics SAS, 75008 Paris, France

**Keywords:** *Danio rerio*, adenovirus, high-capacity adenoviral vector, helper-dependent adenovirus, delivery system, gene therapy

## Abstract

Viral vector use is wide-spread in the field of gene therapy, with new clinical trials starting every year for different human pathologies and a growing number of agents being approved by regulatory agencies. However, preclinical testing is long and expensive, especially during the early stages of development. Nowadays, the model organism par excellence is the mouse (*Mus musculus*), and there are few investigations in which alternative models are used. Here, we assess the possibility of using zebrafish (*Danio rerio*) as an in vivo model for adenoviral vectors. We describe how E1/E3-deleted adenoviral vectors achieve efficient transduction when they are administered to zebrafish embryos via intracranial injection. In addition, helper-dependent (high-capacity) adenoviral vectors allow sustained transgene expression in this organism. Taking into account the wide repertoire of genetically modified zebrafish lines, the ethical aspects, and the affordability of this model, we conclude that zebrafish could be an efficient alternative for the early-stage preclinical evaluation of adenoviral vectors.

## 1. Introduction

Gene therapy seeks to treat or prevent different diseases through the use of genetic materials by following different approaches. Among the best-known, we can highlight the introduction of a new gene to recover a wild-type phenotype (gene supplementation), the correction of an altered genome (gene editing), and the inhibition/inactivation of a mutated gene responsible for a harmful phenotype. To date, there are several mechanisms to introduce the desired genetic material into the target cell, such as physical methods (electroporation, microinjection, etc.), chemical methods (non-viral vectors), and viral vectors [1,2]. Viral vectors are of great clinical interest due to their high efficacy, especially when the genetic material is intended to reach the nucleus. Adenovirus was among the first viruses to be adapted as a gene therapy vector [3]. These non enveloped viruses, with 100 nm icosahedral capsids and linear double-stranded DNA genomes of about 36 Kb, provide remarkable genetic stability and a high efficacy of infection in a variety of cell types. Once inside the cell, viral capsids are degraded in a programmed fashion and lead the genomes into the nucleus, where they stay in an episomal state. Early versions (E1/E3-deleted vectors or first-generation) are useful tools for genetic transfers in vitro, but their in vivo applications are limited to vaccination strategies due to the short duration of transgene expression. This is mainly due to the fact that residual expression of viral genes in transduced cells triggers cytotoxic immune responses [4]. In contrast, third-generation adenoviral vectors, also known as high-capacity vectors (HC-AdV helper-dependent, or “gutless”), are devoid of all viral coding genes, only retaining two *cis*-acting sequences: inverted terminal repeats (ITRs) and packaging signals (ψ) [5]. Apart from possessing an extended cloning capacity, these vectors can sustain transgene expression for long periods of time in vivo in organs with slow cellular turnover, such as the liver or brain [6,7].

Over the years, a wide variety of model organisms have been established for the evaluation of gene therapy vectors. In this sense, the zebrafish has begun to expand with respect to the model organism par excellence—the mouse—due to a series of advantages, such as (i) size, as these animals are around 3 to 4 cm in adulthood, which means less space for maintenance, facilitates manipulation, and allows for the possibility of working with a greater number of individuals at a lower cost; (ii) reproduction, as each couple of zebrafish can produce hundreds of eggs per week, which can be fertilized externally with an embryonic development independent from the mother, allowing analysis from a single-cell stage and follow-up through the whole process [8]. Embryonic and post-embryonic growth is fast, granting the possibility of detecting any anomaly in a matter of days [9]; and (iii) genetics, as zebrafish knock-outs and knock-downs are easy to obtain, and around 70% of human protein coding genes related to diseases have an equivalent gene in this fish, making it possible for researchers to study human diseases on a large scale [10,11]. 

In this work, we describe the transduction efficacy of first- generation and HC-Ad vectors encoding the reporter gene luciferase in zebrafish embryos.

## 2. Materials and Methods

### 2.1. Zebrafish Handling

Adult individuals were held at 28 °C in 30 L aquaria at a rate of 1 fish per liter, with a light–dark cycle of 14:10. The aquarium was located in the veterinary facility of the University of Santiago de Compostela (Lugo), with the REGA code ES270280346401. Embryos came from wild- type individuals, and the moment selected for experimentation corresponded to 48 h post- fertilization (hpf). Every procedure was performed in agreement with the European Parliament and Council Directive 2010/63/EU on the protection of animals used for scientific purposes and the Spain Royal Decree 53/2013 on animal welfare standards. When needed, organisms were euthanized by tricaine (sigma) overdose.

### 2.2. Vectors

All adenoviral vectors were based on human adenovirus type 5 and contained the same expression cassette. It consisted of the CMV enhancer and promoter, the enhanced green fluorescent protein coding sequence (EGFP), and the SV40 polyadenylation sequence. The E1/E3-deleted vector (designated here as Ad-EGFP) has been previously described by the authors of [12]. The HC-AdV (HCA-EGFP) was amplified using the AdTetCre helper virus [13]. Both types of vectors were purified by double- cesium chloride density gradients and desalted by Sephadex size-exclusion columns. The titers of Ad-EGFP and HCA-EGFP were 1.1 × 10^11^ and 8 × 10^10^ infectious units/mL, respectively. Contamination with helper virus was less than 1% in HCA-EGFP.

### 2.3. Microinjection

**Figure 1:**
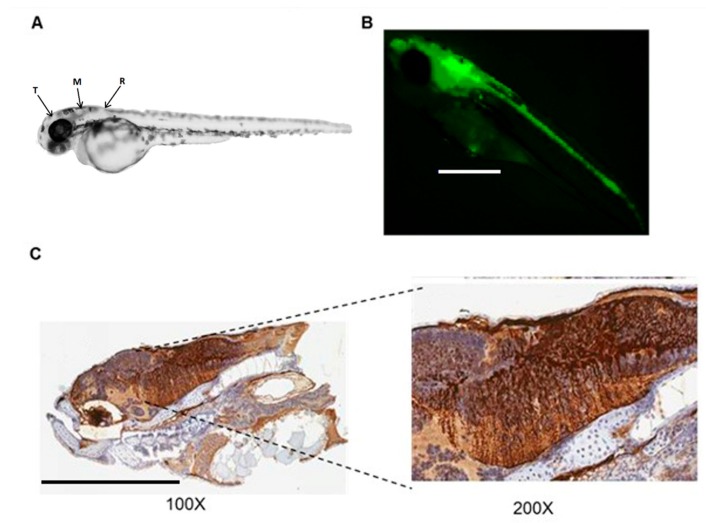
Expression of green fluorescent protein (GFP) in zebrafish embryos injected with Ad-EGFP. (**A**) Schematic representation of brain regions: telencephalon (T), mesencephalon (M), and rhombencephalon (R), modified from. The blue arrow represents the injection area. Ad-EGFP was injected in the brain of zebrafish embryos at 48 hpf. (**B**) Representative images of zebrafish embryos photographed 48 h later using a fluorescence stereomicroscope (100x). (**C**) Immunohistochemistry against GFP (100× and 200× magnification, as indicated).

Zebrafish embryos were anesthetized with 0.3 mg/mL tricaine at 48 h post-fertilization (hpf) before injection. Each vector was loaded into borosilicate glass capillary needles, and the injections were performed using an IM-31 electric microinjector (Narishige). The established parameters’ pressure was 30–35 kPa, with an injection time of 20–30 ms. Injections were executed manually into the midbrain area between the telencephalon and the rhombencephalon, as shown in Figure 1A. The injected volume ranged from 1 to 2 nL.

### 2.4. Incubation

After injections, embryos were placed in two different conditions. First, they were incubated in 60 mm × 15 mm petri dishes with salt dechlorinate tap water obtained from a reverse osmosis filter system for 72 h in 34 °C chambers. Later, positive individuals were transferred to separate aquarium fish tanks, depending on the vector, with the previously explained conditions.

### 2.5. Imaging

Embryos were photographed individually with an AZ-100 Nikon fluorescence stereomicroscope every 24 h post- injection (hpi) in order to visualize the fluorescence progression over time. Images were finally analyzed with ZFTool software (own analysis software is currently being further developed and tested, https://gitlab.citius.usc.es/zebrafish/zftool.) and Photoshop (version CS6, Adobe Inc. Mountain View, CA, USA) in order to compare the fluorescent-positive individuals between different treatments.

Zebrafish embryos showed autofluorescence around the yolk area in the surrounding wavelengths of GFP (Green fluorescent Protein) so in order to accurately discriminate between positive and negative populations, images were also compared with non-injected wild- type controls.

### 2.6. Immunohistochemistry (IHC)

Tissue samples were fixed in 4% formaldehyde (Panreac, Barcelona, Spain) for 24 h, and then incubated in 70% ethanol for an additional 24 h before paraffin embedding. Serial paraffin sections (3-µm thick) were cut, and immunohistochemistry was applied using rabbit anti-GFP (ab6556, Abcam, Cambridge, UK) as the primary antibody (1:2000). Detection was performed by EnVision anti-rabbit K4011 (Dako, Glostrup, Denmark).

### 2.7. Statistical Analysis

Differences observed between the two vectors were analyzed via statistics using PSPP software and Microsoft Excel (version 2010, Microsoft Corporation, Albuquerque, NM, USA). A student’s *t*-test with 95% confidence intervals and a Fisher’s exact test with two tails were applied. Results with *p*-value over 0.05 were considered significant.

## 3. Results

### 3.1. Adenoviral Vectors Transduce the Brain of Zebrafish Embryos

Taking into account that E1/E3-deleted adenoviral vectors are easy to produce at high titers and they share the same tropism as HC-AdV, these are the vectors of choice for an initial assessment of vector transduction and biodistribution. Therefore, we injected the Ad-EGFP vector into the midbrain area between the telencephalon and the rhombencephalon of zebrafish embryos at 48 hpf, and they were firstly visualized using a fluorescent stereomicroscope at 48 hpi. Clear GFP signal was detected in 56% of embryos (Table 1), concentrated in the cephalic region (Figure 1).

**Figure 2:**
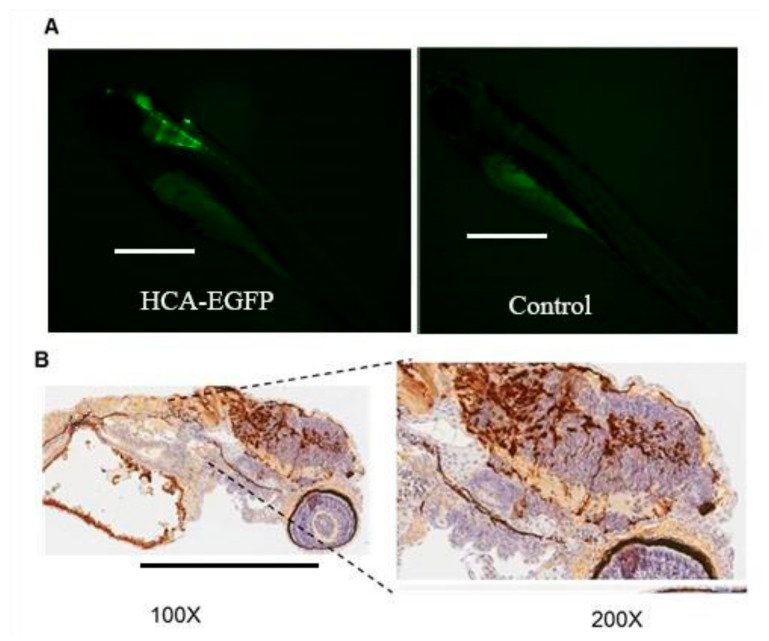
Expression of GFP in zebrafish embryos injected with HCA-EGFP. HCA-EGFP was injected in the brain of zebrafish embryos at 48 hpf. (**A**) Representative images of zebrafish embryos photographed 48 h later using a fluorescence stereomicroscope. A control embryo not injected with the vector is included to show autofluorescence (100x). (**B**) Immunohistochemistry against GFP (100× and 200× magnification, as indicated).

Similar results were obtained when the HCA-EGFP vector was injected in zebrafish embryos using the same experimental conditions (Figure 2). Although the intensity of fluorescence and the percentage of GFP-positive embryos at 48 hpi (36%) were slightly lower than in the case of Ad-EGFP, this quantitative difference could be due to the fact that the HCA-EGFP batch was less concentrated (37% reduction compared to Ad-EGFP). The same trend was observed when the number of positive embryos was quantified at 72 hpi (Table 1). This was the moment in which the fish were moved from petri dishes with salt dechlorinated tap water to the aquarium.

**Figure 3:**
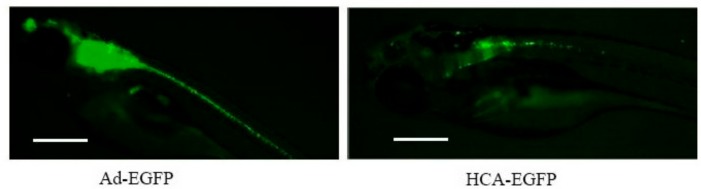
Biodistribution of adenoviral vectors in zebrafish. The indicated vectors were injected in the brain of zebrafish embryos at 48 hpf. Representative images at 3 dpi using a fluorescence stereomicroscope (100x).

The results of immunohistochemistry (Figure 1C and Figure 2B) offered another perspective of the transduced areas. Positive sections matched the surrounding regions of the telencephalon, medulla oblongata, and semicircular canal. Positive spots were detected close to the heart and surrounding the yolk that had not been detected with the fluorescence microscope in the IHC controls. The results were similar for both vectors, although Ad-EGFP showed a greater concentration of positive cell populations along the hindbrain. Fluorescence microphotographs obtained at 3 dpi allowed better visualization of extracranial positive regions close to the heart, extending in a caudal direction (Figure 3).

### 3.2. Hc-Advs Are Better Tolerated Than e1/e3-Deleted Vectors in Zebrafish

Initial evaluation of the mortality rate was performed at 72 hpi, right before the change from petri dishes with salt dechlorinated tap water to the aquarium. This was a stressful process that tended to increase mortality, so it should not be taken into account when evaluating vector-related toxicity. Acute mortality was not statistically different between HCA-EGFP (14%) and Ad-EGFP (18%), despite the higher transduction rate obtained with the latter (Table 2). This mortality rate could have been due to the injection procedure [14], since similar rates were routinely observed when the same volume of vehicle or fluorescent tracer was injected (not shown).

In sharp contrast with this observation, no individuals treated with Ad-EGFP survived past 11 dpi, whereas 36% of zebrafish treated with HCA-EGFP reached the end of the 35- day observation period (Table 2).

### 3.3. Hc-Ad Vectors Allow Sustained Expression of Transgenes in Zebrafish

**Figure 4:**
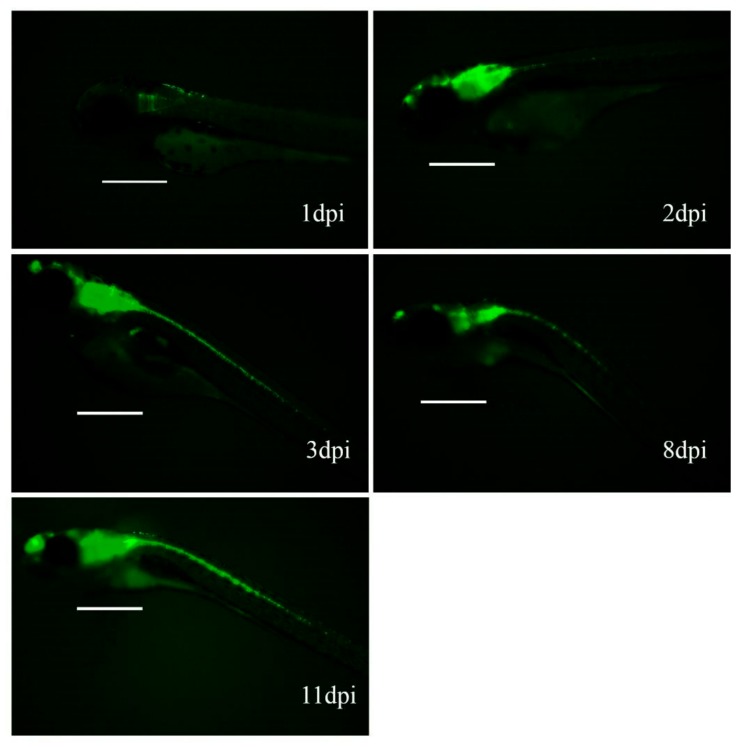
Fluorescence progression in zebrafish injected with Ad-EGFP. The vector was injected in the brain of zebrafish embryos at 48 hpf. Scale = 500 μm.

After an initial maintenance period of 3 dpi and incubation in petri dishes with salt dechlorinated tap water at 34 °C, individual zebrafish were transferred to the aquarium for up to 1 month, with periodic GFP expression reviews. Fluorescence intensity in fish injected with Ad-EGFP increased during the first week (Figure 4), but long-term follow-up was not possible due to the mortality rate observed at 11 dpi.

**Figure 5:**
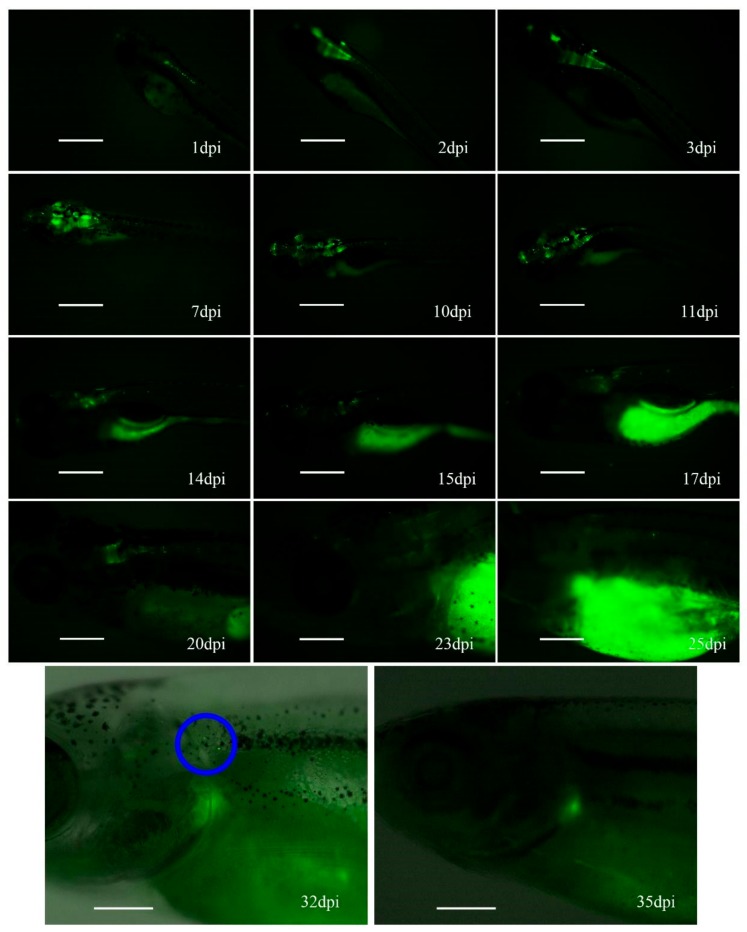
Fluorescence progression in zebrafish injected with HCA-EGFP. The vector was injected in the brain of zebrafish embryos at 48 hpf. The last two images were edited with Photoshop by overlaying a black and white image, facilitating the visualization of the zebrafish. The blue circle marks the alleged cell population remaining positive at 32 dpi, which were absent three days later. Scale = 500 μm.

In contrast, some fish injected with HCA-EGFP completed the 35-day observation period and showed an increase in GFP signal intensity during the first week, followed by a stabilization period, and then finally a decline starting at 15 dpi. The remaining fluorescence completely disappeared at 30 days post-injection (Figure 5).

## 4. Discussion

Since both E1/3-deleted and HC-Ad vectors (Ad-EGFP and HCA-EGFP, respectively) were designed with the same promoter and capsid, equivalent levels of expression and targets would be expected. Despite this, although tropism was similar, the rate of transduced individuals was statistically different, being higher for those treated with Ad-EGFP, with a *p*-value of 0.05. A possibility that arose was that this variability was due to different concentrations for each vector and the fact that the microinjector worked with a fixed pressure instead of a fixed volume. In addition, it should be taken into account that the intensity of GFP expression in transduced cells could be influenced by the vector genome. The strong viral enhancers present in the E1/E3-deleted vectors could increase expression of the transgene, in contrast to the HC-Ad vectors. Stronger expression in cells infected with Ad-EGFP could facilitate the visualization in the fluorescence microscope, contributing to the perception of a higher transduction rate.

As for mortality, results showed no difference between treatments at 72 hpi, with a survival rate over 80%. This observation ruled out acute toxicity derived from infection and/or expression of GFP and viral genes. Again, E1/E3-deleted vectors retained most viral genes in their genome, in contrast with HC-Ads. Although transcription of these genes was activated by E1A, residual expression has been described in the absence of the E1 region in mammalian cells [15]. Therefore, under our experimental conditions, cells infected with Ad-EGFP could express adenoviral proteins, plus higher amounts of GFP in comparison with HCA-EGFP. Both elements could compromise the viability of transduced cells. Although they had no apparent impact on the early development of zebrafish embryos, the scenario was quite different when survival was followed long-term. An increased mortality was relatively normal after 72 hpi. This was related to the environment changes and the fact that zebrafish have a high reproductive rate, so it would be expected that, individually, the survival of the embryos would be lower. However, all individuals treated with Ad-EGFP died before 11 dpi and showed more apathetic behaviors with slow movements, being easier to capture overall. In contrast, more than 30% of fish treated with HCA-EGFP completed the observation period of 35 days, and exhibited normal behaviors. There were potential reasons for this difference. It was possible that the high levels of GFP expression in Ad-EGFP-infected cells caused accumulative cell toxicity, which was not evident during the first 72 hpi. In theory, the involvement of cellular immune responses against GFP or viral epitopes being expressed by the cells was unlikely, taking into account that injection was performed at 48 hpf, when the embryos have an immature immune system [16,17]. However, we could not completely rule out the possibility that immune tolerance was not completely established in our assay conditions, and the persistence of antigen expressions triggered immune responses later on. In this regard, the main difference between cells infected with HCA-EGFP and Ad-EGFP was the presence of adenoviral antigens in the latter, which have been described as an excellent target for cytotoxic responses in mammals [4,15]. In summary, cumulative cellular toxicity caused by viral proteins and/or high GFP expression was the most likely explanation for the reduced survival of zebrafish infected with Ad-EGFP, although immune responses against residual viral gene expressions cannot be ruled out. 

Finally, regarding maintenance of transgene expression, the HC-Ad vector established a fluorescence monitoring up to 32 days. That is, for clinical application, re-administrations should be around this period of time, assuming similar cell replication rates in the population of interest. Nevertheless, other studies in rodents, among other mammals, showed long-term expressions beyond one year [6,7]. This could be due to the higher cellular replication rate that embryos have compared to an adult individual, speeding up the dilution of the transgenes. In fact, the loss of expressions matched with the second half of the embryo transition from larva to juvenile (which went from three days post- fecundation to around 30 days post -fecundation) [18], a period where they also increased significantly in size and morphology, supports this explanation.

## 5. Conclusions

In conclusion, this study demonstrates that adenoviral vectors can transduce zebrafish and maintain expression of transgenes for up to one month (HC-AdV) after a single administration. The small size of the organisms facilitates not only working on a larger scale with a greater number of individuals in less space, but also obtaining results with smaller sample volumes. Although zebrafish will never be a substitute for other mammalian models when it comes to pre clinical experiments, they can be a powerful tool for investigations in early stages.

## Figures and Tables

**Table 1:** Quantification of zebrafish embryos transduced with adenoviral vectors.

Experiments	48 h Post-Injection (hpi)		72 hpi	
	E1/E3-Deleted Vector (Ad-EGFP)	HC-AdV (HCA-EGFP)	Ad-EGFP	HCA-EGFP
Positive	45 (45.92%)	29 (31.87%)	55 (56.12%)	35 (38.46%)
Negative	38 (38.77%)	52 (57.14%)	26 (26.53%)	44 (48.35%)
Dead *	15 (15.30%)	10 (10.99%)	17 (17.35%)	12 (13.19%)
Total	98	91	98	91

Dead *: in reference to those individuals found dead before being introduced to the aquarium tank (<72 hpi).

**Table 2:** Mortality rates in zebrafish embryos injected intracranially with adenoviral vectors at 48 hpf.

Vector	Mortality		
	11 dpi		
	Alive	Dead	Percentage
Ad-EGFP	0	55	100%
HCA-EGFP	12	21	64%
